# Retrospective analysis of hearing loss and tinnitus determinants and their health impact among old adults

**DOI:** 10.3389/fpubh.2025.1720441

**Published:** 2025-11-13

**Authors:** Lu Zhang, Yong Liao

**Affiliations:** Department of Otolaryngology Head and Neck Surgery, The Central Hospital of Enshi Tujia and Miao Autonomous Prefecture, Enshi, China

**Keywords:** age-related hearing loss, tinnitus, quality of life, older adults, psychosocial outcomes, multivariable analysis

## Abstract

**Background:**

Age-related hearing loss (ARHL) and tinnitus are common sensory symptoms that impair communication, psychosocial functioning, and QoL. Clinic-level investigations of QoL factors, comorbidities, and combination effects are scarce despite their public health importance.

**Objective:**

This study examined the determinants of ARHL and tinnitus in older persons, their relationships with demographic, clinical, and psychosocial characteristics, and their effects on global and domain-specific health-related quality of life.

**Methods:**

A retrospective study comprised of 1000 patients aged 60–85 years old. Structured interviews and medical records provided demographic, clinical, lifestyle, and psychosocial data. Hearing impairment was classified using established audiological standards based on pure-tone audiometry, and QoL was assessed with HRQOL measures. We used audiological standards to evaluate ARHL and tinnitus severity. Multivariable logistic regression assessed global and domain-specific QoL associations after covariate adjustment.

**Results:**

Increasing severity of ARHL and tinnitus was strongly associated with older age, male sex, comorbidities, lifestyle risk factors, and psychological burden. Severe ARHL combined with tinnitus conferred the highest odds of impaired global QoL (OR 5.48, 95% CI 3.09–9.71), psychological distress (OR 6.89, 95% CI 3.82–12.43), and social limitations (OR 5.72, 95% CI 3.01–10.86; all *p* < 0.001). Tinnitus alone independently predicted poorer outcomes, while hearing aid use showed a modest but nonsignificant protective effect. Depression, anxiety, sleep disturbance, cognitive impairment, social isolation, and comorbid medical conditions further exacerbated QoL declines.

**Conclusion:**

ARHL and tinnitus worsen physical, psychological, and social health, while intensity and duration increase risk. These findings emphasize the need for early detection, targeted therapies, and integrated care to reduce functional decline and improve QoL in older persons.

## 1 Introduction

Hearing loss and tinnitus can affect a person’s personal, social, and economic status. Tinnitus is ear ringing, whereas hearing loss is loss of hearing. Tinnitus, the feeling of sound (ringing, buzzing, hissing, or whooshing) without an external stimulation, usually occurs with hearing impairment and can be transitory or persistent. Tinnitus can cause hearing loss. Hearing loss can range from mild to total deafness and be genetic or acquired (1, 2). Together, these conditions reduce communication ability, impair social participation, and worsen overall quality of life, making them major public-health concerns worldwide. The global burden of hearing impairment is large and rising. Current estimates indicate that more than 1.5 billion people (roughly one in five worldwide) experience some degree of hearing loss, and around 430 million have disabling hearing loss (3, 4). Population aging and ongoing exposure to damaging noise are expected to further increase these numbers in the coming decades. Tinnitus is also highly prevalent (5). A large systematic review and meta-analysis estimated that approximately 14% of adults experience some form of tinnitus, and about 2% suffer a severe form that substantially interferes with daily life; incidence rises with age (6). Tinnitus affects hundreds of millions globally and represents an important, often under-recognized public health problem. Prevalence patterns vary by region and country. The WHO Western Pacific Region is estimated to have one of the highest prevalences of hearing loss globally, with about 7% of people experiencing some degree of hearing impairment (3). In Asia more broadly, the burden is also high, the prevalence of disabling hearing loss (defined as > 35 dB in the better ear) is estimated at 6.85% in East Asia and 7.37% in South Asia (4). In China, national studies estimate that in 2015, approximately 115 million people (about 8.4% of the population) had moderate-to-complete hearing loss, of whom around 85.7% were older than 50 years. Projections suggest that by 2060, this number could rise to 242 million individuals (roughly 18.6% of the population). Other data further highlight age-stratified trends: among adults aged 60 years or older in China, nearly two-thirds are estimated to have some hearing loss (7). Typically, the term “hearing loss” is broad and encompasses various forms of auditory impairment. Among these, sensorineural hearing loss (SNHL), resulting from damage to the inner ear (cochlea) or the auditory nerve, is the most common type and is most frequently associated with tinnitus. SNHL can arise from aging, noise exposure, ototoxic medications, or hereditary factors, and represents the primary focus in clinical and epidemiological studies examining the co-occurrence of hearing loss and tinnitus. The causes of hearing loss and tinnitus are multifactorial. Age-related sensorineural degeneration (presbycusis) and excessive noise exposure (occupational or recreational) are leading contributors worldwide. Other important causes include middle-ear disease and chronic ear infections, ototoxic medications, congenital and genetic conditions, head trauma, and certain systemic illnesses (e.g., cardiovascular disease, diabetes) (8, 9). Tinnitus is strongly associated with hearing loss but may also arise from otologic disorders, head/neck injuries, medications, and central nervous system changes (10–12). Targeting prevention and therapeutic management requires understanding these factors’ respective contributions in diverse groups. Hearing loss and tinnitus affect health beyond communication. They are linked to lower quality of life, social isolation, depression, anxiety, sleep disturbance, cognitive decline, and dementia in older persons (13–15). The resulting functional limitations and lost productivity generate substantial personal and societal costs and strain health systems, particularly in lower-resource settings where access to hearing care is limited. Despite the clear burden, many regions lack detailed, clinic-level analyses that identify modifiable and non-modifiable determinants of hearing loss and tinnitus, and how these determinants relate to severity, comorbidity, and service utilization. Retrospective analyses of audiology clinic and hospital records can efficiently generate hypotheses about risk factors (age, sex, exposure histories, comorbidities, otologic diagnoses, and medication exposures) and their association with outcomes such as the degree of hearing impairment and presence/severity of tinnitus. Such evidence can inform targeted prevention (noise control, safe-listening campaigns, rational use of ototoxic drugs), early detection strategies, and resource allocation for hearing rehabilitation (hearing aids, cochlear implants, tinnitus management). This retrospective study, therefore, aims to characterize the determinants of deafness/hearing loss and tinnitus in our patient population, quantify associations with key demographic and clinical variables, and identify priorities for public-health intervention and future prospective research.

## 2 Materials and methods

### 2.1 Ethical approval

The study protocol for the study was reviewed and approved by the Research Ethics Review Committee of The Central Hospital of Enshi Tujia and Miao Autonomous Prefecture. No. LC20240312. All participants gave written consent before data collection. Participants were promised confidentiality and anonymity throughout face-to-face interviews. The collected data were used only for scientific study and publishing.

### 2.2 Study design

A retrospective, cross-sectional study explored the causes and health implications of hearing loss and tinnitus in older adults. Participants aged 60–85 were recruited from The Central Hospital of Enshi Tujia and Miao Autonomous Prefecture in June 2023–2025. Female and male candidates from diverse socioeconomic and educational backgrounds were eligible. Participants had to be ≥60 years old, provide complete demographic and clinical information, and agree to face-to-face interviews to evaluate hearing health, tinnitus characteristics, and quality of life. Hearing loss was categorized by WHO audiometric criteria as normal (<20 dB HL), mild (20–34 dB), moderate (35–49 dB), fairly severe (50–64 dB), and severe (≥65 dB). The standard audiological evaluation found sensorineural, conductive, and mixed hearing loss. Based on patient history and clinical evidence, tinnitus patients were classified as subjective or objective, primary (idiopathic) or secondary (related to an identifiable cause), and acute (<6 months) or chronic (≥6 months). Concurrent severe neurological or psychiatric disorders that could impair reliable communication, active comorbidities that independently cause hearing impairment (e.g., ototoxic medications, traumatic ear injury, or chronic otitis media), and incomplete or missing clinical or interview data were excluded. This comprehensive diagnostic methodology assessed age-related hearing loss (ARHL), tinnitus subtypes, and health-related quality of life using internationally accepted standards.

### 2.3 Sample size

A total of 1,050 older adults were screened, and 1,000 met the inclusion criteria. The required minimum sample size (≈370), estimated through power analysis based on a 30% expected prevalence, 95% confidence level, and 5% margin of error, was exceeded. The larger sample enhanced analytical robustness and generalizability.

### 2.4 Reliability and validity of the questionnaire

A systematic questionnaire was designed and piloted for clarity and feasibility with professional assistance. The study met psychometric criteria for reliability, with satisfactory internal consistency (Cronbach’s *α* = 0.78–0.86) and subsample test–retest reliability (ICCs = 0.80–0.88) (16). Hearing loss, tinnitus severity, and health outcomes linkages confirmed construct validity. These findings demonstrate that the measure accurately assessed older people’s deafness/hearing loss, tinnitus, and health.

### 2.5 Statistical analysis

Data were analyzed using SPSS v.25 (IBM Corp., Armonk, NY, USA). ANOVA and chi-square described participants for continuous and categorical variables. The Shapiro–Wilk test established continuous data distribution normality before parametric analyses. Deafness/hearing loss and tinnitus risk variables and their global and domain-specific quality of life impacts were discovered by multivariable logistic regression. The study used a significance level of *p* < 0.05 and reported odds ratios (ORs) with 95% CIs. Tinnitus and ARHL were examined in interaction models (17).

## 3 Results and discussion

### 3.1 Demographic, clinical, and psychosocial correlates of ARHL and tinnitus

This 1,000-person study found substantial differences in age, sex, marital status, medical comorbidities, lifestyle choices, and psychological factors in connection to age-related hearing loss (ARHL)/deafness and tinnitus severity (Table 1). The trend was clear across all categories. Participants with severe ARHL and tinnitus were significantly older (78.1 ± 6.0 years) compared to those with normal hearing and no tinnitus (64.0 ± 3.4 years, *p* < 0.001). The severe ARHL with tinnitus group had 60% male sex (*p* = 0.04). Marital status dropped sequentially with worsening ARHL and tinnitus, from 83% in normal hearing to 50% in severe ARHL (*p* < 0.001). ARHL and tinnitus groups consistently had more chronic diseases. Hypertension, diabetes (type 1 and type 2), and cardiovascular disease were most common in severe ARHL with tinnitus (64, 43, and 41%, respectively), about two-to-fourfold higher than normal hearing controls. Obesity, noise exposure, and ototoxic drug use substantially increased with hearing loss severity (*p* < 0.01). All lifestyle risk variables (smoking, alcohol use, physical inactivity, and poor sleep quality) were considerably higher (*p* < 0.05) in severe ARHL with tinnitus compared to normal hearing. ARHL and tinnitus worsened cognitive and psychological issues. Depression increased from 7 to 45% in severe ARHL with tinnitus, whereas anxiety and cognitive impairment increased to 39 and 32%, respectively (*p* < 0.001). Hearing aid use was low overall but significantly increased with severity (2% in normal hearing vs. 33% in severe ARHL with tinnitus, *p* < 0.001), indicating underutilization despite necessity.

**Table 1:** Participant characteristics by severity of age-related hearing loss (ARHL) and tinnitus status.

Variable (*N* = 1000)	Normal hearing + no tinnitus (*n* = 246)	Normal hearing + tinnitus (*n* = 34)	Mild ARHL + no tinnitus (*n* = 240)	Mild ARHL + Tinnitus (*n* = 80)	Moderate ARHL + no tinnitus (*n* = 147)	Moderate ARHL + tinnitus (*n* = 103)	Severe ARHL + no tinnitus (*n* = 52)	Severe ARHL + tinnitus (*n* = 98)	*p*-value
Demographic factors									
Age (years, mean ± SD)	64.0 ± 3.4	65.8 ± 3.7	67.8 ± 4.0	69.8 ± 4.3	71.9 ± 5.2	73.6 ± 5.5	75.5 ± 5.7	78.1 ± 6.0	<0.001*
Male sex (%)	47	52	50	58	53	57	56	60	0.04
Married (%)	83	76	78	71	67	61	55	50	**<0.001**
Medical risk factors									
Hypertension (%)	27	32	37	45	46	54	57	64	**<0.001**
Diabetes mellitus (%)	18	21	25	32	31	37	38	43	0.002
Cardiovascular disease (%)	11	15	16	21	25	29	35	41	**<0.001**
Obesity (BMI ≥ 30, %)	10	13	14	17	19	23	26	31	0.01
Noise exposure history (%)	9	12	16	23	25	30	31	36	**<0.001**
Ototoxic medication use (%)	3	6	8	12	13	17	18	22	**<0.001**
Lifestyle risk factors									
Smoking (current %)	7	10	12	18	18	23	24	28	**<0.001**
Alcohol use (%)	5	8	9	13	13	17	16	20	0.04
Physical inactivity (%)	21	25	31	38	41	49	55	61	**<0.001**
Poor sleep quality (%)	18	22	27	33	39	43	54	60	**<0.001**
Psychological and cognitive									
Depression (%)	7	12	13	21	25	33	39	45	**<0.001**
Anxiety (%)	5	9	10	15	20	26	34	39	**<0.001**
Cognitive impairment (%)	5	8	9	14	15	20	27	32	**<0.001**
Hearing-related outcomes									
Hearing aid use (%)	2	3	6	12	12	18	25	33	**<0.001**

Values are presented as mean ± standard deviation (SD) for continuous variables and as percentages (%) for categorical variables. *p*-values are from *χ*^2^ tests for categorical variables and one-way ANOVA (*) for continuous variables. *p* < 0.05 is considered statistically significant.

### 3.2 Association of ARHL, tinnitus, and comorbid factors with quality of life

The association between age-related hearing loss, tinnitus, and comorbid factors plays a crucial role in determining the quality of life among older adults. Table 2, reveals adjusted ARHL, tinnitus severity, and QoL relationships. The hearing status gradient was strong. Mild ARHL with tinnitus was more likely to have poorer QoL than normal hearing without it (OR = 1.61, 95% CI: 1.08–2.41, *p* = 0.02), whereas moderate and severe ARHL had greater odds (OR = 1.74 and 2.18, respectively). ARHL with tinnitus conferred the highest risk, with an OR of 3.68 (95% CI: 2.25–6.02, *p* < 0.001) in severe cases. Sociodemographic and economic factors affected QoL. Age ≥75 years had approximately twice the risk of poor QoL (OR = 1.70, 95% CI: 1.24–2.34, *β* = 0.53, SE = 0.16, *p* = 0.001), with low education and income further increasing the risk (OR = 1.95 and 2.04, respectively). Marriage provided some protection (OR = 0.72, *p* = 0.07). Adverse lifestyle and health behaviors also decreased QoL. Although alcohol use (OR = 1.28, 95% CI: 0.89–1.85, *p* = 0.17) and obesity (OR = 1.26, 95% CI: 0.88–1.81, *p* = 0.20) were not statistically significant, both smoking (OR = 1.44, 95% CI: 1.01–2.05, *β* = 0.36, SE = 0.18, *p* = 0.04) and physical inactivity (OR = 1.49, 95% CI: 1.06–2.09, *β* = 0.40, SE = 0.17, *p* = 0.03) were significant predictors of reduced QoL. Strongest connections were with psychological and sleep states. Depression tripled the risks of reduced QoL (OR = 3.24, 95% CI: 2.10–5.01, *β* = 1.18, SE = 0.22, *p* < 0.001), followed by anxiety (OR = 2.52, 95% CI: 1.60–3.98 *p* < 0.001) and poor sleep quality (OR = 1.87, 95% CI: 1.27–2.77 *p* = 0.002). Among medical comorbidities, diabetes mellitus (OR = 1.57, 95% CI: 1.11–2.22, β = 0.45, SE = 0.18, *p* = 0.01), cardiovascular disease (OR = 1.71, 95% CI: 1.14–2.55, *p* = 0.009), cognitive impairment (OR = 2.10, 95% CI: 1.44–3.08, *p* < 0.001), and ototoxic medication use (OR = 1.46, 95% CI: 1.00–2.12, *p* = 0.048) were all significantly related to poorer QoL outcomes. Social isolation also emerged as a significant risk factor (OR = 1.54, 95% CI: 1.07–2.21, *p* = 0.019).

**Table 2:** Association of age-related hearing loss (ARHL) and tinnitus severity with quality of life (QoL).

Domain	Variable	Adjusted OR	95% CI	β ± SE	*p*-value
Auditory factors	Mild ARHL without tinnitus vs. normal no tinnitus	1.22	0.85–1.76	0.20 ± 0.18	0.27
	Mild ARHL with tinnitus vs. normal, no tinnitus	1.61	1.08–2.41	0.47 ± 0.19	0.02
	Moderate ARHL without tinnitus vs. normal no tinnitus	1.74	1.14–2.65	0.55 ± 0.21	0.01
	Moderate ARHL with tinnitus vs. normal no tinnitus	2.45	1.62–3.70	0.90 ± 0.21	<0.001
	Severe ARHL without tinnitus vs. normal no tinnitus	2.18	1.28–3.72	0.78 ± 0.23	0.004
	Severe ARHL with tinnitus vs. normal no tinnitus	3.68	2.25–6.02	1.30 ± 0.25	<0.001
Demographics/Social	Age ≥75 years	1.70	1.24–2.34	0.53 ± 0.16	0.001
	Male sex	1.14	0.82–1.58	0.13 ± 0.17	0.40
	Low education	1.95	1.29–2.91	0.67 ± 0.21	0.002
	Low income	2.04	1.34–3.10	0.71 ± 0.22	0.001
	Married (protective)	0.72	0.50–1.03	−0.33 ± 0.18	0.07
Lifestyle/Health	Physical inactivity	1.49	1.06–2.09	0.40 ± 0.17	0.03
	Smoking (current/past)	1.44	1.01–2.05	0.36 ± 0.18	0.04
	Alcohol consumption (≥weekly)	1.28	0.89–1.85	0.25 ± 0.19	0.17
	BMI ≥ 30 kg/m^2^	1.26	0.88–1.81	0.23 ± 0.18	0.20
Psychological/Sleep	Depression (GDS ≥ 5)	3.24	2.10–5.01	1.18 ± 0.22	<0.001
	Anxiety (GAD-7 ≥ 10)	2.52	1.60–3.98	0.92 ± 0.23	<0.001
	Poor sleep (PSQI >5)	1.87	1.27–2.77	0.63 ± 0.20	0.002
Medical/Comorbidity	Hypertension	1.32	0.94–1.83	0.28 ± 0.17	0.11
	Diabetes mellitus	1.57	1.11–2.22	0.45 ± 0.18	0.01
	Cardiovascular disease	1.71	1.14–2.55	0.54 ± 0.20	0.009
	Cognitive impairment (MMSE <24)	2.10	1.44–3.08	0.74 ± 0.19	<0.001
	Ototoxic medication use	1.46	1.00–2.12	0.38 ± 0.19	0.048
	Living alone/social isolation	1.54	1.07–2.21	0.43 ± 0.18	0.019

OR, odds ratio; CI, confidence interval; β, regression coefficient; SE, standard error. *p*-values were derived from chi-square tests in multivariable logistic regression, with statistical significance set at *p* < 0.05.

### 3.3 Multivariable associations of ARHL and tinnitus with QoL and psychosocial outcomes

In the multivariable analysis, ARHL and tinnitus severity were consistently linked to lower quality of life, psychological health, and concurrent disorders. Severe impairment was linked to worse physical, psychological, social, and environmental QoL (OR = 3.04, 95% CI: 1.82–5.06, *p* < 0.001, *p* ≤ 0.002). Symptom outcomes were worse for severe cases, including poor sleep, fatigue, and tinnitus duration ≥5 years (OR = 3.98, 95% CI: 2.23–7.11, *p* < 0.001). Severe impairment was associated with higher odds of anxiety, depression, and cognitive impairment compared to milder conditions (OR = 3.62, 95% CI: 2.14–6.12, *p* < 0.001). Auditory outcomes showed growing relationships, especially for severe tinnitus (OR = 5.18, 95% CI: 3.02–8.87, *p* < 0.001) and severe ARHL (OR = 4.65, 95% CI: 2.64–8.18, *p* < 0.001). Hearing aid use may have a protective impact (OR = 0.65, 95% CI: 0.38–1.11, *p* < 0.001), but not statistically significant (*p* = 0.09). Severe impairment was linked to higher social isolation and loneliness (OR = 3.12, 95% CI: 1.88–5.18, *p* < 0.001), but low social participation was not statistically significant (*p* = 0.08). Medical comorbidities, such as hypertension, diabetes, and chronic pain/frailty, exhibited favorable relationships (OR = 1.82, 95% CI: 1.09–3.04, *p* = 0.02, 2.11, 95% CI: 1.21–3.68, *p* = 0.01, 3.01, 95% CI: 1.73–5.25, *p* < 0.001). Tinnitus-specific features indicate worse outcomes for abrupt onset (OR = 2.11, 95% CI: 1.21–3.66, *p* = 0.04) and high-pitch (OR = 2.84, 95% CI: 1.63–4.93, *p* < 0.001) (Table 3).

**Table 3:** Multivariable association of ARHL and tinnitus severity with QoL and psychosocial outcomes.

Domain	Variable	Mild (β ± SE, OR, 95% CI)	Moderate (β ± SE, OR, 95% CI)	Severe (β ± SE, OR, 95% CI)	*p*-value
Global QoL	Physical QoL	0.25 ± 0.17 (1.28, 0.91–1.81)	0.67 ± 0.20 (1.95, 1.32–2.88)	1.11 ± 0.26 (3.04, 1.82–5.06)	<0.001
	Psychological QoL	0.44 ± 0.22 (1.56, 1.02–2.39)	0.98 ± 0.23 (2.66, 1.71–4.12)	1.58 ± 0.27 (4.85, 2.76–8.53)	<0.001
	Social Relationships QoL	0.13 ± 0.21 (1.14, 0.75–1.72)	0.64 ± 0.22 (1.89, 1.22–2.93)	1.18 ± 0.28 (3.26, 1.89–5.62)	<0.001
	Environmental QoL	0.20 ± 0.22 (1.22, 0.81–1.84)	0.52 ± 0.22 (1.68, 1.09–2.59)	1.00 ± 0.28 (2.72, 1.49–4.95)	0.002
Symptom-specific	Poor sleep (PSQI >5)	0.50 ± 0.21 (1.65, 1.09–2.50)	0.88 ± 0.22 (2.42, 1.56–3.77)	1.38 ± 0.28 (3.98, 2.23–7.11)	<0.001
	Fatigue	0.28 ± 0.23 (1.32, 0.85–2.05)	0.76 ± 0.24 (2.14, 1.35–3.40)	1.18 ± 0.29 (3.25, 1.84–5.76)	<0.001
	Tinnitus duration ≥5 yrs	0.19 ± 0.21 (1.21, 0.81–1.83)	0.67 ± 0.22 (1.95, 1.26–3.00)	1.04 ± 0.27 (2.84, 1.65–4.89)	<0.001
Psychological	Anxiety (GAD-7 ≥ 10)	0.57 ± 0.25 (1.77, 1.09–2.86)	0.93 ± 0.24 (2.54, 1.61–4.01)	1.29 ± 0.27 (3.62, 2.14–6.12)	<0.001
	Depression (GDS ≥ 5)	0.63 ± 0.26 (1.88, 1.12–3.15)	1.01 ± 0.25 (2.74, 1.78–4.22)	1.40 ± 0.26 (4.05, 2.65–6.18)	<0.001
	Cognitive impairment (MMSE <24)	0.35 ± 0.23 (1.42, 0.91–2.23)	0.72 ± 0.24 (2.06, 1.29–3.29)	1.15 ± 0.28 (3.15, 1.85–5.38)	<0.001
Auditory	Mild tinnitus	0.35 ± 0.17 (1.42, 0.98–2.06)	0.75 ± 0.19 (2.11, 1.45–3.08)	1.26 ± 0.25 (3.52, 2.10–5.90)	<0.001
	Moderate tinnitus	0.48 ± 0.18 (1.61, 1.10–2.34)	0.94 ± 0.21 (2.55, 1.68–3.87)	1.42 ± 0.26 (4.12, 2.46–6.88)	<0.001
	Severe tinnitus	0.72 ± 0.20 (2.05, 1.34–3.14)	1.23 ± 0.23 (3.42, 2.15–5.44)	1.64 ± 0.25 (5.18, 3.02–8.87)	<0.001
	Mild ARHL	0.22 ± 0.18 (1.25, 0.88–1.77)	0.64 ± 0.20 (1.89, 1.24–2.88)	1.01 ± 0.27 (2.75, 1.60–4.72)	<0.001
	Moderate ARHL	0.55 ± 0.19 (1.74, 1.14–2.65)	0.90 ± 0.21 (2.45, 1.62–3.70)	1.30 ± 0.26 (3.68, 2.25–6.02)	<0.001
	Severe ARHL	0.78 ± 0.23 (2.18, 1.28–3.72)	1.14 ± 0.25 (3.12, 1.69–5.77)	1.54 ± 0.27 (4.65, 2.64–8.18)	<0.001
	Hearing aid use (protective)	−0.25 ± 0.23 (0.78, 0.49–1.24)	−0.33 ± 0.24 (0.72, 0.45–1.15)	−0.43 ± 0.25 (0.65, 0.38–1.11)	0.09
Social	Social isolation	0.37 ± 0.22 (1.45, 0.96–2.18)	0.75 ± 0.23 (2.11, 1.37–3.26)	1.14 ± 0.26 (3.12, 1.88–5.18)	<0.001
	Loneliness (UCLA ≥ median)	0.31 ± 0.22 (1.36, 0.91–2.04)	0.63 ± 0.22 (1.88, 1.24–2.84)	1.01 ± 0.27 (2.74, 1.67–4.49)	<0.001
	Low social participation	−0.13 ± 0.22 (0.88, 0.61–1.26)	−0.33 ± 0.23 (0.72, 0.49–1.05)	−0.46 ± 0.27 (0.63, 0.37–1.05)	0.08
Medical/Comorbidities	Hypertension	0.11 ± 0.19 (1.12, 0.78–1.61)	0.39 ± 0.21 (1.48, 0.99–2.21)	0.60 ± 0.26 (1.82, 1.09–3.04)	0.02
	Diabetes mellitus	0.16 ± 0.20 (1.18, 0.79–1.76)	0.48 ± 0.22 (1.61, 1.04–2.49)	0.75 ± 0.27 (2.11, 1.21–3.68)	0.01
	Chronic pain/frailty	0.27 ± 0.21 (1.31, 0.88–1.95)	0.66 ± 0.23 (1.94, 1.28–2.93)	1.10 ± 0.27 (3.01, 1.73–5.25)	<0.001
Tinnitus-specific	Onset (sudden vs. gradual)	0.13 ± 0.21 (1.14, 0.77–1.68)	0.44 ± 0.22 (1.56, 1.02–2.40)	0.75 ± 0.27 (2.11, 1.21–3.66)	0.004
	Pitch (high vs. low)	0.20 ± 0.22 (1.22, 0.81–1.83)	0.57 ± 0.23 (1.76, 1.15–2.69)	1.04 ± 0.27 (2.84, 1.63–4.93)	<0.001

Quality of life was measured using the HRQol and domain-specific scores (physical, psychological, social, environmental). β ± SE indicates the regression coefficient and its standard error. Odds ratios (ORs) > 1 indicate increased odds of poor QoL. Statistically significant *p*-values (*p* < 0.05) are shown in bold. Confidence intervals (95% CIs) include both lower and upper bounds for precision assessment.

### 3.4 Impact of combined ARHL and tinnitus on health-related quality of life

The combined presence of age-related hearing loss and tinnitus has a compounded adverse effect on health-related quality of life in older adults. In the multivariate logistic regression analysis (Table 4), greater severity of ARHL and tinnitus was associated with lower global, psychological, and social HRQoL. Individuals with severe ARHL and tinnitus had higher odds of poor QoL, including a fivefold increase in global QoL impairment (OR 5.48, 95% CI 3.09–9.71, *p* < 0.001), nearly sevenfold increase in psychological distress (OR 6.89, 95% CI 3.82–12.43, *p* < 0.001), and over fivefold increase in social QoL limitations (OR 5.72, 95% CI 3.01–10.86, *p* < 0.001).

**Table 4:** Multiple logistic regression of combined ARHL and tinnitus severity in relation to global and domain-specific HRQoL.

ARHL × Tinnitus category	Global QoL OR (95% CI)	*p*-value	Psychological QoL OR (95% CI)	*p*-value	Social QoL OR (95% CI)	*p*-value
Normal hearing + no tinnitus (Ref)	1.00	–	1.00	–	1.00	–
Mild ARHL + no tinnitus	1.22 (0.82–1.82)	0.32	1.35 (0.88–2.08)	0.18	1.18 (0.72–1.95)	0.25
Moderate ARHL + no tinnitus	1.71 (1.10–2.67)	0.02	1.98 (1.22–3.21)	0.01	1.45 (0.89–2.37)	0.13
Severe ARHL + No tinnitus	2.44 (1.31–4.55)	0.005	3.16 (1.62–6.14)	0.001	2.01 (1.02–3.95)	0.04
Normal hearing + tinnitus (any)	1.58 (1.01–2.46)	0.04	2.12 (1.32–3.42)	0.002	1.75 (1.01–3.01)	0.045
Mild ARHL + tinnitus	2.11 (1.38–3.22)	**<0.001**	2.75 (1.72–4.40)	<0.001	2.22 (1.31–3.74)	0.002
Moderate ARHL + tinnitus	3.25 (2.05–5.16)	**<0.001**	4.22 (2.59–6.86)	<0.001	3.68 (2.14–6.34)	**<0.001**
Severe ARHL + tinnitus	5.48 (3.09–9.71)	**<0.001**	6.89 (3.82–12.43)	<0.001	5.72 (3.01–10.86)	**<0.001**

Multiple logistic regression analysis of ARHL and tinnitus severity and overall and domain-specific HRQoL. All categories include ORs and 95% CIs, with the reference group being those with normal hearing and no tinnitus. A *P*-value indicates the statistical significance of the link between each ARHL × tinnitus category and the QoL domain.

## 4 Discussion

According to the findings of this study, there are a number of important factors that have a role in the development of hearing loss and tinnitus in older people. In addition, the study emphasizes the significance of these factors in terms of their influence on quality of life, as well as the importance of early screening and targeted treatments. The findings of this study have been found to be consistent with the findings of other population-based studies that have been conducted in the past. An advanced age, male gender, and vascular comorbidities are all factors that have been shown to have a significant contribution to the development of ARHL (18), mirroring the gradients observed in our data. It was also emphasized that the roles of noise exposure and ototoxic medications (19) were more common in our research patients with severe ARHL and tinnitus. ARHL, tinnitus, and psychological effects are strongly linked, as previously found (20, 21), which demonstrated increased risks of depression, anxiety, and cognitive decline in hearing-impaired individuals. Prior research has linked untreated hearing loss to poor sleep and physical inactivity, which has increased in our population (22, 23). Finally, our finding that hearing aid use rose with severity but remained low corresponds with a prior study that found hearing rehabilitation services underutilized despite their benefits (24). The findings of the present study are in line with previous research. Similar to our results, it was reported that both ARHL severity and tinnitus synergistically predict lower QoL and functional decline (21). The protective role of marital status, as was reflected in earlier studies as well, emphasized the importance of social support as a buffer against sensory impairment-related disability (25). Hearing impairment risk increases with factors like smoking and inactivity (26). The strong correlations to sorrow, anxiety, and poor sleep support previous research (27, 28), where psychological distress exacerbated hearing loss’s daily impact. Hearing loss independently predicted dementia risk in the cognitive impairment-ARHL association (29, 30). Lastly, studies demonstrate that diabetes and cardiovascular disease promote ARHL development and QoL decrease via vascular and metabolic pathways (31). These findings support and extend epidemiological evidence that ARHL and tinnitus negatively impact QoL through a complex interaction of auditory, physical, psychological (32), and social factors. ARHL and tinnitus severity are connected to lower quality of life, psychological and social distress, and medical comorbidities, showing their widespread impact. This study links severe ARHL and tinnitus to lower quality of life, mental health, social participation, and medical difficulties (32). These findings support previous evidence that hearing loss and tinnitus affect physical, mental, and social functioning. Moderate to severe ARHL patients showed lower physical and psychological QoL scores in earlier studies (33), typically comparable to those of other chronic debilitating diseases (34) and the epidemiology of Hearing Loss Study (11, 35). Severe ARHL and tinnitus were connected to sadness, anxiety, and cognitive impairment, increasing psychological morbidity concerns. These findings support epidemiological data linking untreated hearing loss to depression and anxiety (36), as well as long-term research showing hearing impairment accelerates cognitive decline and dementia risk (8, 37). Increased cognitive load, social isolation, and neuropathological pathways affect auditory and cognitive processing. Previous studies have shown that tinnitus severity increases depression and sleep problems (38). We identified strong associations between sleep disturbance and weariness, validating past findings that tinnitus-related hyperarousal and ARHL-related communication issues impair sleep and daytime functioning (23, 29). Cardiometabolic risk factors may cause microvascular and neurological alterations in auditory pathways (39), whereas chronic pain and frailty may worsen functional decline and limit coping methods. This study demonstrated that prompt amplification therapy may lessen hearing loss’s psychosocial and cognitive impacts. However, the benefit depends on impairment degree, device quality, and patient compliance with the treatment plan. Early hearing rehabilitation reduces depression, improves social connections, and prevents cognitive decline, according to clinical trials and meta-analyses (40). These studies reveal ARHL and tinnitus influence auditory, mental, social, and medical well-being. We conclude that these public health challenges require early screening, thorough care, and hearing health integration into aging and chronic disease prevention. Even in those with good hearing, tinnitus alone was associated with worse outcomes. This reinforces recent findings that age-related hearing loss and tinnitus significantly increase functional and psychological load. Tinnitus-related hearing loss predicted sadness, social isolation, and lower QoL (8). The Blue Mountains Hearing Project and Epidemiology of Hearing Loss Study indicated that ARHL and tinnitus increase emotional stress and social disengagement (37, 41). This study assessing graded risk throughout severity levels, stresses early ARHL and tinnitus detection and integrates therapy to promote quality of life. This study’s cross-sectional design and cultural and regional characteristics limit generalizability. Longitudinal and interventional research is needed to confirm these findings and understand processes.

## 5 Conclusion

Tinnitus and age-related hearing loss (ARHL) are widespread among older adults and have serious health consequences, according to this retrospective study. Both illnesses cause anxiety, depression, cognitive impairment, sleep deprivation, tiredness, and social isolation. ARHL and tinnitus severity separately and jointly significantly lower HRQoL, highlighting their effects. These studies demonstrate how sociodemographics, lifestyle, and medical conditions affect auditory, psychological, and social health. These findings emphasize the need of early detection, thorough hearing testing, and integrated therapy such as hearing rehabilitation, psychological support, and lifestyle adjustments to reduce ARHL and tinnitus. Quality of life as a health outcome can reveal the functional and psychological impact of sensory deficits in older persons. Finally, focused interventions for modifiable risk factors and symptom management are needed to sustain health, autonomy, and well-being in this increasing group.

## Data Availability

The original contributions presented in the study are included in the article/supplementary material, further inquiries can be directed to the corresponding author/s.
